# Supplementary material for: Invisible attitudes have visible effects on the behaviour of animals in scientific studies
Source: Anim Cogn. 2026 Jul 10;29(1):68. doi: 10.1007/s10071-026-02085-x (PMC13638684; doi:10.1007/s10071-026-02085-x)
Supplement: Supplementary file 1 [file 10071_2026_2085_MOESM3_ESM.docx]

**Supporting Information for**

Invisible attitudes have visible effects on the behavior of animals in scientific studies

Svenja Capitain^1^*, Gwendolyn Wirobski^1,2^, Catherine-Noémie Alexandrina Guran^3,4^, Andreas Berghänel^5^, Claus Lamm^3^, Sarah Marshall-Pescini^1^, & Friederike Range^1^*.

^1^ Domestication Lab, Konrad Lorenz Institute of Ethology, Department of Interdisciplinary Life Sciences, University of Veterinary Medicine Vienna, 1210 Vienna, Austria

^2^ Comparative Cognition Group, Institute of Biology, Faculty of Science, Université de Neuchâtel, 2000 Neuchâtel, Switzerland

^3^ Social, Cognitive and Affective Neuroscience Unit, Department of Cognition, Emotion, and Methods in Psychology, Faculty of Psychology, University of Vienna, 1010 Vienna, Austria

^4^ Institute of Cognitive and Affective Neuroscience, Department of Psychology, Medical School Hamburg, 20457 Hamburg, Germany

^5^ Department of Zoology and Animal Ecology, Institute of Biology and Chemistry, University of Hildesheim, 31141 Hildesheim, Germany

*Svenja Capitain, Friederike Range

**Email:** Svenja.capitain@vetmeduni.ac.at, friederike.range@vetmeduni.ac.at

**This PDF file includes:**

Supporting text

Figures S1 to S4

Legends for Movies S1

Legends for Datasets S1

**Other supporting materials for this manuscript include the following:**

Movies S1

Datasets S1

Table of contents

[Supporting Material Files	3](#_Toc232703687)

[Explicit questionnaire development	3](#_Toc232703688)

[Specifics on the Implicit Association Task	4](#_Toc232703689)

[Ethogram	6](#_Toc232703690)

[Trainers	6](#_Toc232703691)

[Animals	6](#_Toc232703692)

[Interrater reliability	7](#_Toc232703693)

[Trainers	7](#_Toc232703694)

[Animals	8](#_Toc232703695)

[PCA	8](#_Toc232703696)

[Human PCA	8](#_Toc232703697)

[Animal PCA	9](#_Toc232703698)

[Frequentist results	9](#_Toc232703699)

[Q1: How are explicit attitude, implicit attitude and experience related?	9](#_Toc232703700)

[Explicit	9](#_Toc232703701)

[Implicit	10](#_Toc232703702)

[Q2: Do attitudes influence human physiology and behavior?	10](#_Toc232703703)

[Heart rate	10](#_Toc232703704)

[Heart rate variability	10](#_Toc232703705)

[Cortisol	11](#_Toc232703706)

[Human PC1: Ease of Contact	11](#_Toc232703707)

[Human PC2: Open Talking	11](#_Toc232703708)

[Q 3-a: Does human attitude influence animal behavior?	12](#_Toc232703709)

[Animal PC1: Affiliation	12](#_Toc232703710)

[Animal PC2: Interest	12](#_Toc232703711)

[Animal PC3: Discomfort	12](#_Toc232703712)

[Q 3-b: Does human physiology influence animal behavior?	13](#_Toc232703713)

[Animal PC1: Affiliation	13](#_Toc232703714)

[Animal PC2: Interest	13](#_Toc232703715)

[Animal PC3: Discomfort	14](#_Toc232703716)

[Check: Human Behavior -> HR	14](#_Toc232703717)

[Check: Human Behavior -> HRV	14](#_Toc232703718)

[Check: Human Behavior -> Cortisol	14](#_Toc232703719)

[Bayesian results	15](#_Toc232703720)

[Q1: How are explicit attitude, implicit attitude and experience related?	15](#_Toc232703721)

[Explicit	15](#_Toc232703722)

[Implicit	16](#_Toc232703723)

[Q2: Do attitudes influence human physiology and behavior?	16](#_Toc232703724)

[Heart rate	16](#_Toc232703725)

[Heart rate variability	17](#_Toc232703726)

[Cortisol	17](#_Toc232703727)

[Human PC1: Ease of Contact	18](#_Toc232703728)

[Human PC2: Open Talking	18](#_Toc232703729)

[Q 3-a: Does human attitude influence animal behavior?	19](#_Toc232703730)

[Animal PC1: Affiliation	19](#_Toc232703731)

[Animal PC2: Interest	20](#_Toc232703732)

[Animal PC3: Discomfort	20](#_Toc232703733)

[Q 3-b: Does human physiology influence animal behavior?	21](#_Toc232703734)

[Animal PC1: Affiliation	21](#_Toc232703735)

[Animal PC2: Interest	21](#_Toc232703736)

[Animal PC3: Discomfort	22](#_Toc232703737)

[Time series PCA	23](#_Toc232703738)

[Human PCA	23](#_Toc232703739)

[Animal PCA	23](#_Toc232703740)

[Time series output	24](#_Toc232703741)

[Human influence on PC1_Animal_Affiliation	26](#_Toc232703742)

[Human influence on PC2_Animal_Interest	27](#_Toc232703743)

[Human influence on PC3_Animal_Discomfort	28](#_Toc232703744)

[Animal influence on PC1_Human_ActiveContact	29](#_Toc232703745)

[Animal influence on PC2_Human_OpenPosture	30](#_Toc232703746)

[Animal influence on PC3_Human_Direction	32](#_Toc232703747)

## Supporting Material Files

- **Movie S1 (separate file). An overview over the test procedure with dogs and wolves respectively.**
- **Dataset S1 (separate file). The data underlying the analyses. The metadata is presented in the first slide, the full data file is presented in the second slide.**

# Explicit questionnaire development

The questionnaire was developed as part of a research project on attitudes towards dogs and wolves. For this study, we extracted explicit attitude from the questions on positive affect, negative affect, and concerns towards dogs and wolves respectively. The questions were rated on a Likert scale from 1 (strongly disagree) to 4 (strongly agree). Negative questions were reverse coded for analysis. Which animal appeared first in the questionnaire was counterbalanced. Because our study group was biased towards dogs and wolves given their chosen profession, we evaluated the internal consistency of various item combinations. Items whose inclusion reduced or did not contribute to Cronbach’s alpha were excluded (n=5), resulting in a twelve-item scale with the highest internal consistency (α = 0.78 for wolves; α = 0.57 for dogs). The final item list is provided below. The mean rating across the 12 items was calculated for each handler and species as a measure of explicit attitude of that handler towards dogs and wolves respectively.

**The final 12-item list comprising the explicit attitude score:**

1. It is dangerous to meet a dog/wolf outside
2. To me dogs/wolves seem unpredictable.
3. The dog/wolf is a dangerous animal.
4. It is dangerous for an adult to meet a dog/wolf.
5. It is dangerous for a small pet (guinea pig, rabbit, cat) to meet a dog/wolf.
6. It is dangerous for a dog to meet another dog/ a wolf.
7. It is dangerous for a sheep to meet a dog/wolf.
8. Dogs/Wolves have emotions just like us.
9. I would help an injured dog/wolf.
10. I would pay money for the treatment of an injured dog/wolf.
11. Dogs/Wolves should be treated with as much respect as humans are.
12. One should care for a dog/wolf in the same way as for people.

# Specifics on the Implicit Association Task

The Implicit Association Task (IAT) was programmed in Python. At the start of the task, the handler received written instructions on using the E key to classify stimuli to the left and the I key to classify stimuli to the right in correspondence with the categories displayed in the upper corners. They were also asked to complete the task as quickly and correctly as possible. The IAT was comprised of seven blocks of trials. The stimuli were presented individually in the centre of the computer monitor in randomized order. As stimuli, ten pictures of dogs and wolves respectively (**Figure S1**; matched in body posture and valence) were used, with word stimuli comprising ten positive (“harmless, kind, relaxed, friendly, good, cute, clever, good-natured, mild”) and ten negative adjectives (“aggressive, frightening, hostile, mean, evil, dangerous, creepy, dark, threatening, cold”).

***Figure S1: The picture stimuli used in the Implicit Association Task.***

For an overview of the block structure, see **Figure S2**. In the first block, the stimuli comprised the twenty dog and wolf pictures with the word “Dogs” and “Wolves” in the upper left and right corner for half the participants, and in the right and left corner for the other half. If the participant made an incorrect choice, a red cross appeared, and they had to correct their choice before the next picture appeared. In the second block, the stimuli comprised ten positive and ten negative adjectives, with the categories “Positive” and “Negative” in the upper corners respectively, again counterbalanced by participants and with feedback if the choice was incorrect. In the third block, the categories of both previous blocks appeared in the previously trained corners together (e.g. Dog and Positive in the left, and Wolf and Negative in the right corner), and twenty random trials of dog pictures, wolf pictures, negative words and positive words followed, again with feedback. Finally, in block four, the same paradigm as in block three was repeated, but with forty trials and without feedback. In block five, the same paradigm as in block one (just dog and wolf pictures) took place, but the category corners were switched and twenty trials with feedback took place to train the reversal. Block six and seven were equivalent to block three and four, but with the categories of dog and wolf switched (following the previous example, now Wolf and Positive were in the left, and Dog and Negative in the right corner). Thus, half of the trainers first had Dog and Positive paired, while the other half started with Wolf and Positive. Further, half the participants had the Positive pair in the right corner, and the other half in the left corner.

**Figure S2: A presentation of the Implicit Association Test block structure.**

To measure the implicit bias for each participant following Greenwald, Nosek & Banaji (2003)^[1]^, we calculated the mean response time in trials three, four, six and seven. The difference in the mean response time of trial three and six as well as trial four and seven was divided by their combined standard deviation respectively. After correcting for whether participants had Dog and Positive or Wolf and Positive in the first test trials, the mean between the two resulting scores represented the D1 score, ranging from -1 to 1. Therein, negative scores correspond to faster responses when wolves were paired with positive and dogs with negative words, suggesting the person unconsciously paired wolves much more with positive words and dogs more with negative words. Positive scores suggest the opposite pattern, and a person that unconsciously paired dogs with more positive words than wolves.

# Animal testing table

***Table S1:*** *An overview over the number of tests per animal and trainer. Each canid is identified by name and species (W=wolf, D=dog, m=male, f=female), the trainers are identified by number. If a trainer and a dog or wolf interacted, it is identified as “yes”. If they did not, it is identified as “no” and the reason is given in brackets. The number of dogs/wolves and trainers each trainer/canid interacted with, is given in the Total columns. The total number of interactions across all animals and trainers is calculated in the bottom right corner.*

|  | | Trainer | | | | | | | | |  |
| --- | --- | --- | --- | --- | --- | --- | --- | --- | --- | --- | --- |
|  |  | 1 | 2 | 3 | 4 | 5 | 6 | 7 | 8 | 9 | Total (animal) |
| Animal | Enzi (Dm) | yes | yes | yes | yes | yes | yes | yes | yes | yes | 9 |
|  | Imara (Df) | yes | yes | yes | yes | yes | yes | yes | yes | yes | 9 |
|  | Panya (Df) | yes | yes | yes | yes | yes | yes | yes | yes | yes | 9 |
|  | Hiari (Dm) | yes | yes | yes | yes | yes | yes | yes | yes | yes | 9 |
|  | Tala (Wf) | yes | yes | yes | yes | yes | yes | yes | yes | No (not trained) | 8 |
|  | Una (Wf) | yes | yes | yes | yes | yes | yes | yes | yes | No (not trained) | 8 |
|  | Amarok (Wm) | yes | yes | yes | yes | yes | yes | yes | yes | No (not trained) | 8 |
|  | Wamblee (Wm) | yes | yes | yes | yes | yes | yes | yes | No (died) | No (not trained) | 7 |
|  | Total (trainer) | 4W 4D | 4W 4D | 4W 4D | 4W 4D | 4W 4D | 4W 4D | 4W 4D | 3W 4D | 0W  4D | 67 |

# HRV correction method

Using a script written in RStudio, the time between heartbeats in milliseconds (RR intervals) were screened for artifacts using a threshold-based method(42). Intervals deviating more than 150 ms from the median RR were flagged as artifacts. Identified artifacts were corrected by cubic spline interpolation between adjacent valid intervals (or replaced by the median if occurring at the first position).

# Ethogram

## Trainers

| **Behavior** | | **Description** |
| --- | --- | --- |
| Gazing at the animal (D) | | Trainer’s head is directed towards the animal while the animal is visible |
| **Body language** | | |
|  | Angled towards animal (D) | The torso (irrespective of the head) is angled towards the (visible) animal |
|  | Ration closed/open body posture | Open = the person does not actively place something (e.g., own body) between themselves and the animal (hands in pockets counts as open unless it’s the vest pockets and the arms are drawn to the front)  Closed = the person places items or their arms between themselves and the animal (e.g., crossed or clasped hands, arm across body, crossed legs…)   - Unless it is just to quickly reach across in the pocket (esp. training), but it is closed if the hand stays there   !!! Only measured when the torso is angled towards the animal |
|  | Self-directed behaviors (D) | Face/Head/Hair touching, rubbing arms, hugging oneself, yawning, fidgeting (e.g., finger rubbing, mindless scratching (scratching without looking there)) |
|  | Repetitive movements (D) | Repetitive movements = continual, discrete behaviors that are repeated rapidly / consistently, such as foot tapping, finger tapping |
|  | Shoulders up (D) | Shoulders pulled up towards ears, not back + down and/or relaxed |
| **Only during cuddling test phase** | | |
|  | Ration increasing/ decreasing distance (D) | Increasing = motions that increase distance: body leaning away from animal (i.e., deviating from a straight axis), pushing animal away, stepping away (e.g., half steps back/side wards), pulling hand away/up when animal gets close  Decreasing = motions that decrease distance: (slight) forward lean (i.e., deviating from straight axis), reaching towards animal, stepping towards them  Only measured when animal within 2 bdl |
|  | Calling by name (F) | Calling the animal by name (Burkhard et al. 2023) |
|  | Talking to the animal (D) | Speech directed towards the animal (apart from calling by name) |
|  | Active touching (D) | Time spent actively touching (i.e., reaching out, not animal leaning against them) the animal while the animal is within proximity |
| **Only during training phase – Nothing additional** | | |
| Trial duration (D) | | from stepping two feet into the enclosure until E calls stop |
| Non-visible (D) | | the upper part of the body is not visible |

## Animals

| **Behavior** | **Description** |
| --- | --- |
| Proximity seeking (D) | Proportion of time spent (min. two feet) in proximity (within one body length) of the trainer (Burkhard et al. 2023) |
| Self-directed behaviors (F) | Nose/lip licking = animal extends the tongue to move it over its lips/ nose  Yawning = animal opens the jaw wide without vocalization  Body shake = animal shakes body+head from side to side  Autogrooming/scratching = animal grooms/licks/scratches themselves |
| Gazing at the human (D) | Head oriented towards the human |
| Exploring the human (D) | Sniffing (= nose within 10 cm of human with head directed towards the human), licking (= extended tongue moves along parts of the human) or mouthing (= taking parts of the human into one’s mouth) |
| Submissive (D) | One or more of the following: head lowered with ears pushed back with tail wagging low with low body posture, crouching together with licking (self or others), exposing belly |
| Fear (D) | One or more of the following: crouched with tail between the legs, fleeing (moving away faster than a walk), backing away (walking away backwards), freezing, startle response (=jumping back), risk assessment (i.e. back legs extended, nose reaches towards object) |
| Tail wagging (D) | Animal moves the tail from side to side more so than the movement automatically generated through body movement (e.g. walking) |
| **Only during cuddling test phase** | |
| Physical contact (D) | Time spent in physical contact with the human (both actively (i.e., self-initiated) or passively (i.e., tolerating human touch)) while in proximity (Wirobski et al. 2021) |
| Approach-leave ratio (frequencies) (S) | Approach = animal (min. two paws) comes actively within 1 bdl from the human  Leaving = animal actively moves from within less than 1 bdl to being within more than 1 bdl from the human (i.e., doesn’t count if the human is the one leaving) |
| **Only during training phase** | |
| Commands followed (F) | Ratio of number of times the animal displays the requested command divided by the total number of commands given (for the first four-minute sequence) (Indicate with modifier whether command-followed or command-not followed) |
| “Down”-command followed (F) | Ratio of number of times following the down command (or staying down when the animal rewards them) divided by the total number of commands given -- (indicate with modifier whether followed or not followed) |
| Trial duration (D) | from stepping two feet into the enclosure to E says stop |
| Non-visible (D) | Upper half of the animal is not visible |

# Interrater reliability

## Trainers

|  | **Intraclass correlation coefficient (ICC)** | |
| --- | --- | --- |
| **Behavior** | **Cuddling phase** | **Training phase** |
| Gazing at the animal (D) | 0.93 | 0.87 |
| Angled towards animal (D) | 0.94 | 0.83 |
| Open body posture | 0.72 | 0.92 |
| Closed body posture | 0.75 | 0.92 |
| Self-directed behaviors (D) | 0.61 | 0.84 |
| Repetitive movements (D) | 0.99 | Na |
| Shoulders up (D) | 0.99 | Na |
| Decreasing distance (D) | 0.91 | - |
| Increasing distance (D) | 0.98 | - |
| Calling by name (F) | 0.96 | - |
| Talking to the animal (D) | 0.99 | - |
| Active touching (D) | 0.84 | - |
| **Average by phase** | **0.88** | **0.88** |
| **Average** | **0.88** | |

## Animals

|  | **Intraclass correlation coefficient (ICC)** | |
| --- | --- | --- |
| **Behavior** | **Cuddling phase** | **Training phase** |
| Proximity seeking (D) | 0.86 | 0.99 |
| Self-directed behaviors (F) |  |  |
| Autogrooming | 0.80 | Na |
| Body shake | 0.92 | Na |
| Nose licks | 0.91 | 0.87 |
| Yawning | 0.91 | Na |
| Gazing at the human (D) | 0.95 | 0.97 |
| Exploring the human (D) | 0.92 | - |
| Submissive (D) | 0.99 | Na |
| Fear (D) | 0.81 | 0.99 |
| Tail wagging (D) | 0.74 | 0.80 |
| Physical contact (D) | 0.89 | - |
| Approaching (F) | 0.85 | - |
| Leaving (F) | 0.91 | - |
| Commands followed (F) | - | 1 |
| “Down”-command followed (F) | - | 0.96 |
| **Average by phase** | **0.88** | **0.92** |
| **Average** | **0.90** | |

# PCA

Based on the summed duration of each respective behavior across the first (I_) and second (T_) phase of the test session.

## Human PCA

**Eigenvalues:**

|  | **Eigenvalue** | **Variance (%)** | **Cumulative variance (%)** |
| --- | --- | --- | --- |
| **Dim.1: PC1-Ease of Contact** | 2.25 | 30.75 | 30.75 |
| **Dim.2: PC2-Open Talking** | 1.27 | 17.38 | 48.14 |
| Dim.3 | 1.10 | 15.05 | 63.19 |
| Dim.4 | 0.82 | 11.27 | 74.46 |
| Dim.5 | 0.61 | 8.32 | 82.77 |
| Dim.6 | 0.52 | 7.06 | 89.84 |

- ***Note***: only the first two were retained due to model complexity and explanatory validity

**Loadings:**

|  | **Dim.1** PC1-Ease of Contact | **Dim.2** PC2-Open Talking |
| --- | --- | --- |
| C _ActiveTouch_Aprox_Dur_z | **0.81** | -0.02 |
| C _BodyAngledToAnimal_Avisible_Dur_z | **0.63** | -0.02 |
| C _DecreasingDistance_Aprox_Dur_z | **-0.75** | 0.37 |
| C_Stress_RepMov.ShoulersUp.SelfDirect_SumFreq_zlog | **-0.57** | -0.08 |
| C_TalkingToTheAnimal_Dur_z | 0.01 | **0.83** |
| T_BodyAngledToAnimal_Avisible_Dur_z | **0.19** | -0.11 |
| T_GazingAtAnimal_Avis_Dur_z | **0.23** | -0.07 |
| C_Op.ClosedBody_Dur_Ratio_zlog | 0.34 | **0.58** |
| T_Op.ClosedBody_Dur_Ratio_zlog | 0.30 | **0.30** |

## Animal PCA

**Eigenvalues:**

|  | **eigenvalue** | **Variance (%)** | **Cumulative variance (%)** |
| --- | --- | --- | --- |
| **Dim.1: PC1-Affiliation** | 4.75 | 34.96 | 34.96 |
| **Dim.2: PC2-Interest** | 1.87 | 13.79 | 48.75 |
| **Dim.3: PC3-Discomfort** | 1.32 | 9.70 | 58.46 |
| Dim.4 | 1.15 | 8.45 | 66.91 |
| Dim.5 | 0.96 | 7.09 | 74.00 |
| Dim.6 | 0.91 | 6.67 | 80.67 |

**Loadings:**

|  | **Dim.1** PC1-Affiliation | **Dim.2** PC2-Interest | **Dim.3** PC3-Discomfort |
| --- | --- | --- | --- |
| C _ApproachLeave_Ratio_z | **0.56** | -0.25 | -0.34 |
| Both_Stress_GroomShake.Yawn_whileVis_SumFreq_z | 0.01 | 0.06 | **0.64** |
| C _ExploringHum_whileProx_z | -0.40 | **0.70** | -0.32 |
| Both_FearSubmission_SumFreq_z | -0.16 | 0.06 | **0.50** |
| C _GazingHum_whileVis_Dur_z | 0.02 | **0.83** | 0.16 |
| C _NoseLicks_Freq_zlog | **0.71** | 0.11 | 0.09 |
| C _PhysicalContact_whileProx_Dur_z | **0.68** | -0.41 | 0.11 |
| C _Proximity_Dur_z | **0.75** | -0.21 | 0.14 |
| C_Tailwag_whileVis_Dur_z | **0.53** | 0.50 | 0.29 |
| T_CommandFollowed_AbsFreq_z | **0.79** | 0.16 | 0.06 |
| T_DownCommad_followed_AbsFreq_z | **0.63** | 0.25 | -0.25 |
| T_DownCommad_Ratio_z | **0.51** | 0.19 | -0.45 |
| T_GazingHum_whileVis_Dur_z | **0.71** | 0.10 | 0.01 |
| T_Proximity_Dur_z | **0.62** | 0.09 | 0.00 |
| T_Tailwag_whileVis_Dur_z | **0.51** | 0.02 | 0.14 |

# Frequentist results

## Q1: How are explicit attitude, implicit attitude and experience related?

### Explicit

Expl_model.red3 <- glmmTMB(Explicit_scaled ~ Species*IAT_D1Score.z + Experience_years.z +

(1|Trainer), family=beta_family(link = "logit"), data=Edata)

**Full-null comparison**:

|  | Df | AIC | BIC | logLik | deviance | Chisq Chi | Df | p-value |  |
| --- | --- | --- | --- | --- | --- | --- | --- | --- | --- |
| Expl_model.0 | 3 | -20.72 | -18.22 | 13.36 | -26.72 |  |  |  |  |
| Expl_model | 8 | -20.81 | -14.15 | 18.41 | -36.81 | 10.09 | 5 | 0.07 | . |

**Summary**:

|  | Estimate | Std. Error | z-value | p-value |  | Upper  95% CI | Lower 95% CI |
| --- | --- | --- | --- | --- | --- | --- | --- |
| (Intercept) | 1.52 | 0.28 | 5.50 | 0.00 | *** | 1.00 | 2.15 |
| SpeciesWolf | -0.46 | 0.21 | -2.15 | 0.03 | * | -0.88 | -0.08 |
| IAT_D1Score.z | -0.09 | 0.29 | -0.32 | 0.75 |  | -0.70 | 0.50 |
| Experience_years.z | -0.45 | 0.27 | -1.66 | 0.09 | . | -1.00 | 0.08 |
| SpeciesWolf:IAT_D1Score.z | 0.22 | 0.21 | 1.06 | 0.29 |  | -0.21 | 0.65 |

### Implicit

IAT_model <- glmmTMB(IAT_scaled ~ Experience_years.z,

family=beta_family(link="logit"), data=Idata)

**Full-null comparison**:

|  | Df | AIC | BIC | logLik | deviance | Chisq Chi | Df | p-value |
| --- | --- | --- | --- | --- | --- | --- | --- | --- |
| IAT_model.0 | 2 | 2.26 | 2.65 | 0.87 | -1.74 |  |  |  |
| IAT_model | 3 | 4.01 | 4.60 | 1.00 | -1.99 | 0.25 | 1 | 0.62 |

## Q2: Do attitudes influence human physiology and behavior?

### Heart rate

HR_model<-lme4::lmer(Mean_ChangeHR ~ Species*(IAT_D1Score.z+Explicit.z) +

Experience_years.z + (1|Trainer) + (1|Animal), data=HRdata)

**Full-null comparison**:

|  | npar | AIC | BIC | logLik | Chisq | Df | p-value |
| --- | --- | --- | --- | --- | --- | --- | --- |
| HR_model.0 | 5 | 411.63 | 422.65 | -200.82 |  |  |  |
| HR_model | 10 | 418.07 | 440.12 | -199.03 | 3.56 | 5 | 0.61 |

### Heart rate variability

**Linear-cubic comparison**:

|  | npar | AIC | BIC | logLik | Chisq | Df | p-value |
| --- | --- | --- | --- | --- | --- | --- | --- |
| HRV_model | 8 | 473.01 | 490.65 | -228.51 |  |  |  |
| HRV_quad | 12 | 477.55 | 504.01 | -226.78 | 3.46 | 4 | 0.48 |

HRV_model.REML.red <- lmerTest::lmer(Mean_ChangeRMSSD_corr1 ~

Species+(IAT_D1Score.z + Explicit.z) + Experience_years.z +

(1|Trainer) + (1|Animal), REML=T, data=HRVdata)

**Full-null comparison**:

|  | npar | AIC | BIC | logLik | Chisq | Df | p-value |
| --- | --- | --- | --- | --- | --- | --- | --- |
| HRV_model.0 | 5 | 477.75 | 488.77 | -233.88 | NA | NA | NA |
| HRV_model | 8 | 473.01 | 490.65 | -228.51 | 10.74 | 3 | 0.01 |

**Summary**:

|  | Estimate | Std. Error | df | t-value | p-value | Upper  95% CI | Lower 95% CI |
| --- | --- | --- | --- | --- | --- | --- | --- |
| (Intercept) | 2.97 | 2.00 | 7.92 | 1.49 | 0.17 | -0.61 | 6.67 |
| SpeciesWolf | -5.36 | 2.20 | 43.62 | -2.43 | 0.02 | -10.04 | -0.94 |
| IAT_D1Score.z | -3.75 | 1.77 | 5.31 | -2.11 | 0.08 | -7.22 | 0.05 |
| Explicit.z | -2.37 | 1.88 | 11.00 | -1.26 | 0.23 | -6.18 | 1.39 |
| Experience_years.z | -2.38 | 1.93 | 6.22 | -1.24 | 0.26 | -6.04 | 1.36 |

### Cortisol

Cort_model<-lmer(Cort_Change ~ Species*(IAT_D1Score.z + Explicit) + Experience_years.z +

(1|Trainer) + (1|Animal), data=Cortdata)

**Full-null comparison**:

|  | npar | AIC | BIC | logLik | Chisq | Df | p-value |
| --- | --- | --- | --- | --- | --- | --- | --- |
| Cort_model.0 | 5 | 288.43 | 298.99 | -139.22 |  |  |  |
| Cort_model | 10 | 293.11 | 314.22 | -136.56 | 5.32 | 5 | 0.38 |

### Human PC1: Ease of Contact

PC1H_model.REML.red<-lmerTest::lmer(PC1_Hum_EaseContact ~ Species+(IAT_D1Score.z +

Explicit.z) + Experience_years.z + (1|Trainer) + (1|Animal),

data=PC1Hdata, REML=T)

**Full-null comparison**:

|  | npar | AIC | BIC | logLik | Chisq | Df | p-value |  |
| --- | --- | --- | --- | --- | --- | --- | --- | --- |
| PC1H_model.0 | 5 | 303.33 | 313.97 | -146.67 |  |  |  |  |
| PC1H_model | 10 | 301.73 | 323.00 | -140.86 | 11.61 | 5 | 0.04 | * |

**Summary**:

|  | Estimate | Std. Error | df | t-value | p-value |  | Upper  95% CI | Lower 95% CI |
| --- | --- | --- | --- | --- | --- | --- | --- | --- |
| (Intercept) | 1.91 | 1.06 | 5.99 | 1.80 | 0.12 |  | 0.11 | 3.63 |
| SpeciesWolf | -2.76 | 0.89 | 12.16 | -3.09 | <0.01 | ** | -4.71 | -0.96 |
| IAT_D1Score.z | 0.37 | 0.99 | 4.53 | 0.37 | 0.73 |  | -1.41 | 1.95 |
| Explicit.z | 0.70 | 0.77 | 26.42 | 0.92 | 0.37 |  | -0.67 | 2.19 |
| Experience_years.z | 0.35 | 1.03 | 5.30 | 0.34 | 0.75 |  | -1.46 | 2.22 |

### Human PC2: Open Talking

PC2H_model<-lmer(PC2_Hum_OpenTalking ~ Species*(IAT_D1Score.z + Explicit.z) +

Experience_years.z + (1|Trainer) + (1|Animal), data=PC2Hdata)

**Full-null comparison**:

|  | npar | AIC | BIC | logLik | Chisq | Df | p-value |
| --- | --- | --- | --- | --- | --- | --- | --- |
| PC2H_model.0 | 5 | 190.80 | 201.35 | -90.40 |  |  |  |
| PC2H_model | 10 | 198.88 | 219.99 | -89.44 | 1.91 | 5 | 0.86 |

## Q 3-a: Does human attitude influence animal behavior?

### Animal PC1: Affiliation

**Linear-cubic comparison**:

|  | npar | AIC | BIC | logLik | Chisq | Df | p-value |
| --- | --- | --- | --- | --- | --- | --- | --- |
| PC1A_Att_model | 10 | 304.94 | 326.21 | -142.47 |  |  |  |
| PC1A_Att_quad_I | 12 | 306.04 | 331.56 | -141.02 | 2.9 | 2 | 0.23 |

PC1A_Att_model.REML.red1<-lmerTest::lmer(PC1_Anim_Affiliation ~ Species * (Explicit.z)

+ IAT_D1Score.z +	Experience_years.z + (1|Trainer) + (1|Animal),

data=PC1AttAdata, REML=T)

**Full-null comparison**:

|  | npar | AIC | BIC | logLik | Chisq | Df | p-value |  |
| --- | --- | --- | --- | --- | --- | --- | --- | --- |
| PC1A_Att_model.0 | 5 | 322.21 | 332.85 | -156.11 |  |  |  |  |
| PC1A_Att_model | 10 | 304.94 | 326.21 | -142.47 | 27.27 | 5 | <0.001 | *** |

**Summary**:

|  | Estimate | Std. Error | df | t-value | p-value |  | Upper  95% CI | Lower 95% CI |
| --- | --- | --- | --- | --- | --- | --- | --- | --- |
| (Intercept) | 4.04 | 0.83 | 5.65 | 4.85 | <0.001 | ** | 2.52 | 5.65 |
| SpeciesWolf | -7.33 | 1.20 | 6.59 | -6.09 | <0.001 | *** | -9.79 | -4.79 |
| Explicit.z | 0.24 | 0.51 | 10.25 | 0.46 | 0.65 |  | -0.72 | 1.16 |
| IAT_D1Score.z | -0.12 | 0.37 | 3.41 | -0.33 | 0.76 |  | -0.77 | 0.61 |
| Experience_years.z | 0.82 | 0.41 | 3.75 | 1.98 | 0.12 |  | -0.10 | 1.58 |
| SpeciesWolf:Explicit.z | 1.41 | 0.71 | 46.65 | 2.00 | 0.05 | . | -0.10 | 2.85 |

### Animal PC2: Interest

PC2A_Att_model<-lmer(PC2_Anim_Interest ~ Species * (IAT_D1Score.z + Explicit.z) +

Experience_years.z + (1|Trainer) + (1|Animal), data=PC2AttAdata)

**Full-null comparison**:

|  | npar | AIC | BIC | logLik | Chisq | Df | p-value |
| --- | --- | --- | --- | --- | --- | --- | --- |
| PC2A_Att_model.0 | 5 | 258.18 | 268.81 | -124.09 |  |  |  |
| PC2A_Att_model | 10 | 266.45 | 287.72 | -123.22 | 1.73 | 5 | 0.89 |

### Animal PC3: Discomfort

PC3A_Att_model.REML.red1<-lmerTest::lmer(PC3_Anim_Discomfort ~ Species *Explicit.z

+ IAT_D1Score.z + Experience_years.z + (1|Trainer) + (1|Animal),

REML=T, data=PC3AttAdata)

**Full-null comparison**:

|  | npar | AIC | BIC | logLik | Chisq | Df | p-value |  |
| --- | --- | --- | --- | --- | --- | --- | --- | --- |
| PC3A_Att_model.0 | 5 | 205.53 | 216.17 | -97.77 |  |  |  |  |
| PC3A_Att_model | 10 | 205.91 | 227.18 | -92.95 | 9.63 | 5 | 0.09 | . |

**Summary**:

|  | Estimate | Std. Error | df | t-value | p-value |  | Upper  95% CI | Lower 95% CI |
| --- | --- | --- | --- | --- | --- | --- | --- | --- |
| (Intercept) | -0.27 | 0.49 | 6.13 | -0.54 | 0.61 |  | -1.31 | 0.72 |
| SpeciesWolf | 0.31 | 0.70 | 6.50 | 0.44 | 0.68 |  | -1.11 | 1.73 |
| Explicit.z | 0.46 | 0.22 | 13.98 | 2.03 | 0.06 | . | 0.01 | 0.83 |
| IAT_D1Score.z | 0.09 | 0.16 | 5.03 | 0.54 | 0.61 |  | -0.23 | 0.38 |
| Experience_years.z | 0.24 | 0.18 | 5.50 | 1.31 | 0.24 |  | -0.11 | 0.59 |
| SpeciesWolf:Explicit.z | -0.71 | 0.31 | 47.87 | -2.28 | 0.03 | * | -1.28 | -0.05 |

## Q 3-b: Does human physiology influence animal behavior?

### Animal PC1: Affiliation

PC1A_Phys_model.REML.red1<-lmerTest::lmer(PC1_Anim_Affiliation ~ Species * (Mean_ChangeHR.z

+ Mean_ChangeRMSSD_corr1.z) + Cort_Change.z + Experience_years.z +

(1|Trainer) + (1|Animal), data=PC1PhysAdata, REML=T)

**Full-null comparison**:

|  | npar | AIC | BIC | logLik | Chisq | Df | p-value |  |
| --- | --- | --- | --- | --- | --- | --- | --- | --- |
| PC1A_Phys_model.0 | 5 | 296.92 | 307.04 | -143.46 |  |  |  |  |
| PC1A_Phys_model | 12 | 261.58 | 285.89 | -118.79 | 49.33 | 7 | <0.001 | *** |

**Summary**:

|  | Estimate | Std. Error | df | t-value | p-value |  | Upper  95% CI | Lower 95% CI |
| --- | --- | --- | --- | --- | --- | --- | --- | --- |
| (Intercept) | 4.02 | 0.44 | 2.68 | 9.10 | <0.001 | ** | 3.14 | 4.82 |
| SpeciesWolf | -8.46 | 0.68 | 3.85 | -12.39 | <0.001 | *** | -9.97 | -6.78 |
| Mean_ChangeHR.z | -0.17 | 0.56 | 47.43 | -0.30 | 0.76 |  | -1.29 | 0.79 |
| Mean_ChangeRMSSD_corr1.z | 0.34 | 0.61 | 46.04 | 0.56 | 0.58 |  | -1.18 | 1.65 |
| Cort_Change.z | 0.29 | 0.32 | 47.97 | 0.91 | 0.37 |  | -0.28 | 0.91 |
| Experience_years.z | 0.21 | 0.32 | 42.52 | 0.64 | 0.53 |  | -0.47 | 0.71 |
| SpeciesWolf:Mean_ChangeHR.z | 4.18 | 0.87 | 47.77 | 4.83 | <0.001 | *** | 2.58 | 5.82 |
| SpeciesWolf:Mean_ChangeRMSSD_ corr1.z | 1.75 | 0.83 | 47.01 | 2.12 | 0.04 | * | 0.02 | 3.86 |

### Animal PC2: Interest

PC2A_Phys_model<-lme4::lmer(PC2_Anim_Interest ~ Species * (Mean_ChangeHR.z +

Mean_ChangeRMSSD_corr1.z + Cort_Change.z) + Experience_years.z +

(1|Trainer) + (1|Animal), data=PC2PhysAdata)

**Full-null comparison**:

|  | npar | AIC | BIC | logLik | Chisq | Df | p-value |
| --- | --- | --- | --- | --- | --- | --- | --- |
| PC2A_Phys_model.0 | 5 | 237.42 | 247.55 | -113.71 |  |  |  |
| PC2A_Phys_model | 12 | 244.90 | 269.20 | -110.45 | 6.52 | 7 | 0.48 |

### Animal PC3: Discomfort

PC3A_Phys_model<-lmer(PC3_Anim_Discomfort ~ Species * (Mean_ChangeHR.z +

Mean_ChangeRMSSD_corr1.z + Cort_Change.z) + Experience_years.z +

(1|Trainer) + (1|Animal), data=PC3PhysAdata)

**Full-null comparison**:

|  | npar | AIC | BIC | logLik | Chisq | Df | p-value |
| --- | --- | --- | --- | --- | --- | --- | --- |
| PC3A_Phys_model.0 | 5 | 188.78 | 198.91 | -89.39 |  |  |  |
| PC3A_Phys_model | 12 | 194.35 | 218.66 | -85.18 | 8.42 | 7 | 0.30 |

## Check: Human Behavior -> HR

HR_Behav_model<-lme4::lmer(Mean_ChangeHR ~ Species*(PC2_Hum_OpenTalking.z +

PC1_Hum_EaseContact.z) + Experience_years.z + (1|Trainer) + (1|Animal),

data=HR_Behav_data)

**Full-null comparison**:

|  | npar | AIC | BIC | logLik | Chisq | Df | p-value |
| --- | --- | --- | --- | --- | --- | --- | --- |
| HR_Behav_model.0 | 5 | 377.33 | 387.89 | -183.66 |  |  |  |
| HR_Behav_model | 10 | 384.55 | 405.65 | -182.27 | 2.79 | 5 | 0.73 |

## Check: Human Behavior -> HRV

**Linear-cubic comparison**:

|  | npar | AIC | BIC | logLik | Chisq | Df | p-value |
| --- | --- | --- | --- | --- | --- | --- | --- |
| HRV_Behav_model | 10 | 438.63 | 459.74 | -209.32 |  |  |  |
| HRV_Behav_quad_1 | 12 | 441.73 | 467.06 | -208.87 | 0.90 | 2 | 0.63 |

HRV_Behav_model <- lme4::lmer(Mean_ChangeRMSSD_corr1 ~

Species*(PC1_Hum_EaseContact.z + PC2_Hum_OpenTalking.z) + Experience_years.z +

(1|Trainer) + (1|Animal), data=HRV_Behav_data)

**Full-null comparison**:

|  | npar | AIC | BIC | logLik | Chisq | Df | p-value |
| --- | --- | --- | --- | --- | --- | --- | --- |
| HRV_Behav_model.0 | 5 | 439.12 | 449.68 | -214.56 | NA | NA | NA |
| HRV_Behav_model | 10 | 438.63 | 459.74 | -209.32 | 10.49 | 5 | 0.06 |

**Summary:**

|  | Estimate | Std. Error | df | t-value | p-value | Upper  95% CI | Lower 95% CI |
| --- | --- | --- | --- | --- | --- | --- | --- |
| (Intercept) | 1.35 | 2.45 | 9.03 | 0.55 | 0.59 | -2.94 | 6.83 |
| SpeciesWolf | -2.81 | 2.35 | 54.34 | -1.20 | 0.24 | -7.26 | 1.68 |
| PC1_Hum_EaseContact.z | 1.58 | 1.42 | 55.38 | 1.12 | 0.27 | -1.35 | 4.60 |
| PC2_Hum_OpenTalking.z | 1.00 | 1.07 | 52.76 | 0.93 | 0.36 | -1.09 | 3.13 |
| Experience_years.z | -2.21 | 2.30 | 7.08 | -0.96 | 0.37 | -6.72 | 1.47 |

## Check: Human Behavior -> Cortisol

Cort_Behav_model<-lmer(Cort_Change ~ Species*(PC1_Hum_EaseContact.z +

PC2_Hum_OpenTalking.z) + Experience_years.z + (1|Trainer) + (1|Animal),

data=Cort_Behav_data)

**Full-null comparison**:

|  | npar | AIC | BIC | logLik | Chisq | Df | p-value |
| --- | --- | --- | --- | --- | --- | --- | --- |
| Cort_Behav_model.0 | 5 | 257.75 | 267.79 | -123.88 |  |  |  |
| Cort_Behav_model | 10 | 262.54 | 282.61 | -121.27 | 5.21 | 5 | 0.39 |

# Bayesian results

**Analysis:** For each model, we ran four Markov Chain Monte Carlo (MCMC) chains with 4,000 iterations (1,000 warm-up), yielding 12,000 posterior samples. Weakly informative priors were used for all models and are reported in detail below. Convergence was assessed via effective sample sizes and 𝑅-hat statistics (<1.01). Posterior predictive checks confirmed model fit, and Leave-One-Out cross-validation (LOO) with the null and reduced models were used to decide on the most reliable model. Estimates are reported as posterior means with 95% credible intervals (CrI). Bayes factors were generated based on Savage-Dickey density ratio.

## Q1: How are explicit attitude, implicit attitude and experience related?

### Explicit

**Priors**: prior(normal(0, 0.3), class = "b"), prior(normal(1.1, 0.20), class = "Intercept"), prior(gamma(1, 0.1), class = "phi”))

model_Explicit_unique <- brm(

formula = Explicit_scaled ~ species * (IAT_D1Score.z + Experience_years.z) + (1 | trainer),

data = df_e,

family = Beta(),

prior = priors_Expl,

chains = 4, # more MCMC chains improve robustness of posterior

iter = 4000,

control = list(adapt_delta = 0.8), #delta = acceptance rate (default 80%, increase if no convergence)

seed = 1234,

save_pars = save_pars(all = TRUE))

**Full-null model comparison:**

|  | elpd_diff | se_diff |
| --- | --- | --- |
| model_Explicit_unique | 0 | 0 |
| model_Explicit_null | -0.3 | 0.4 |
| model_Explicit_reduced | -0.5 | 0.8 |

| **Summary**: | Estimate | Est.Error | lower 95% CI | upper 95% CI | Rhat | Bulk_ESS | BF |
| --- | --- | --- | --- | --- | --- | --- | --- |
| Intercept | 1.28 | 0.19 | 0.9 | 1.66 | 1 | 6463 | 42.74 |
| speciesWolf | -0.28 | 0.21 | -0.68 | 0.15 | 1 | 10231 | 1.75 |
| IAT_D1Score.z | -0.03 | 0.22 | -0.46 | 0.4 | 1 | 6053 | 0.73 |
| Experience_years.z | -0.23 | 0.23 | -0.66 | 0.24 | 1 | 5332 | 1.36 |
| speciesWolf:IAT_D1Score.z | 0.08 | 0.2 | -0.31 | 0.49 | 1 | 9678 | 0.74 |
| speciesWolf:Experience_years.z | 0.12 | 0.21 | -0.29 | 0.54 | 1 | 9409 | 0.87 |

### Implicit

**Priors**: prior(normal(0, 0.3), class = "b"), prior(normal(0, 0.20), class = "Intercept"), prior(gamma(1, 0.1), class = "phi")

model_IAT_D1Score_unique <- brm(

formula = IAT_scaled ~ Species*Experience_years.z + (1|trainer),

data = df_i,

family = Beta(),

prior = priors_Impl,

chains = 4,

iter = 4000,

control = list(adapt_delta = 0.95),

seed = 1234,

save_pars = save_pars(all = TRUE))

**Full-null comparison:**

|  | elpd_diff | se_diff |
| --- | --- | --- |
| model_Implicit_null_unique | 0 | 0 |
| model_Implicit_reduced_unique | -1.6 | 0.1 |
| model_IAT_D1Score_unique | -3.3 | 0.3 |

| **Summary**: | Estimate | Est.Error | lower 95% CI | upper 95% CI | Rhat | Bulk_ESS | BF |
| --- | --- | --- | --- | --- | --- | --- | --- |
| Intercept | -0.07 | 0.21 | -0.47 | 0.33 | 1 | 4178 | 0.89 |
| SpeciesWolf | -0.01 | 0.17 | -0.34 | 0.32 | 1 | 7533 | 0.55 |
| Experience_years.z | 0.06 | 0.27 | -0.47 | 0.58 | 1 | 2746 | 0.90 |
| SpeciesWolf:Experience_years.z | 0.01 | 0.15 | -0.29 | 0.32 | 1 | 6367 | 0.50 |

## Q2: Do attitudes influence human physiology and behavior?

### Heart rate

**Priors**: prior(normal(0, 0.5), class = "b"), prior(normal(0, 10), class = "Intercept")

model_HR_reduced <- brm(

formula = Mean_ChangeHR ~ species + (IAT_D1Score.z + Explicit.z) + Experience_years + (1 | trainer) +(1|Animal),

data = df,

family = gaussian(),

prior = priors_HR,

chains = 4,

iter = 4000,

control = list(adapt_delta = 0.8),

seed = 1234)

**Full-null model comparison:**

|  | elpd_diff | se_diff |
| --- | --- | --- |
| model_HR_reduced | 0 | 0 |
| model_HR_null | 0 | 0.3 |
| model_HR | -0.1 | 0.3 |

| **Summary**: | Estimate | Est.Error | lower 95% CI | upper 95% CI | Rhat | Bulk_ESS | BF |
| --- | --- | --- | --- | --- | --- | --- | --- |
| Intercept | -1.97 | 2.86 | -7.39 | 3.89 | 1 | 3676 | 0.19 |
| speciesWolf | 0.14 | 0.49 | -0.85 | 1.09 | 1 | 8769 | 1.00 |
| IAT_D1Score.z | 0.13 | 0.48 | -0.83 | 1.08 | 1 | 8166 | 0.96 |
| Explicit.z | -0.2 | 0.48 | -1.14 | 0.75 | 1 | 8422 | 1.04 |
| Experience_years | 0.27 | 0.31 | -0.38 | 0.85 | 1 | 4922 | 1.01 |

### Heart rate variability

**Priors**: prior(normal(0, 0.5), class = "b"), prior(normal(0, 10), class = "Intercept")

model_HRV_reduced <- brm(

formula = Mean_ChangeRMSSD_corr1 ~ species + (IAT_D1Score.z + Explicit.z) + Experience_years.z + (1 | trainer) +(1|Animal),

data = df,

family = gaussian(),

prior = priors_HRV,

chains = 4,

iter = 4000,

control = list(adapt_delta = 0.8),

seed = 1234)

**Full-null model comparison:**

|  | elpd_diff | se_diff |
| --- | --- | --- |
| model_HRV_reduced | 0 | 0 |
| model_HRV | -0.2 | 0.1 |
| model_HRV_null | -0.3 | 0.2 |

| **Summary**: | Estimate | Est.Error | lower 95% CI | upper 95% CI | Rhat | Bulk_ESS | BF |
| --- | --- | --- | --- | --- | --- | --- | --- |
| Intercept | 0.54 | 2.39 | -4.22 | 5.23 | 1 | 2478 | 0.24 |
| speciesWolf | -0.19 | 0.49 | -1.14 | 0.77 | 1 | 9646 | 1.09 |
| IAT_D1Score.z | -0.2 | 0.49 | -1.19 | 0.77 | 1 | 8881 | 1.08 |
| Explicit.z | 0.07 | 0.48 | -0.89 | 1.01 | 1 | 8374 | 1.01 |
| Experience_years.z | -0.1 | 0.49 | -1.06 | 0.85 | 1 | 10175 | 0.95 |

### Cortisol

**Priors**: prior(normal(0, 0.5), class = "b"), prior(normal(0, 5), class = "Intercept")

model_Cort_reduced <- brm(

formula = Cort_Change ~ species + (IAT_D1Score.z + Explicit.z) + Experience_years.z + (1 | trainer) +(1|Animal),

data = df,

family = gaussian(),

prior = priors_Cort,

chains = 4,

iter = 4000,

control = list(adapt_delta = 0.9),

seed = 1234)

**Full-null model comparison:**

|  | elpd_diff | se_diff |
| --- | --- | --- |
| model_Cort | 0 | 0 |
| model_Cort_reduced | -0.2 | 1 |
| model_Cort_null | -0.4 | 1.6 |

| **Summary**: | Estimate | Est.Error | lower 95% CI | upper 95% CI | Rhat | Bulk_ESS | BF |
| --- | --- | --- | --- | --- | --- | --- | --- |
| Intercept | -0.35 | 0.52 | -1.35 | 0.71 | 1 | 4570 | 0.13 |
| speciesWolf | 0.1 | 0.43 | -0.75 | 0.96 | 1 | 7996 | 0.90 |
| IAT_D1Score.z | -0.02 | 0.31 | -0.62 | 0.58 | 1 | 7898 | 0.68 |
| Explicit.z | -0.06 | 0.32 | -0.7 | 0.56 | 1 | 7026 | 0.71 |
| Experience_years.z | -0.52 | 0.32 | -1.14 | 0.11 | 1 | 6086 | 2.55 |

### Human PC1: Ease of Contact

**Priors**: prior(normal(0, 0.5), class = "b"), prior(normal(0, 2), class = "Intercept")

model_HBehav1_reduced <- brm(

formula = PC1_Hum_EaseContact ~ species + (IAT_D1Score.z + Explicit.z) + Experience_years.z + (1 | trainer) +(1|Animal),

data = df,

family = gaussian(),

prior = priors_HBehav1,

chains = 4,

iter = 4000,

control = list(adapt_delta = 0.9),

seed = 1234)

**Full-null model comparison:**

|  | elpd_diff | se_diff |
| --- | --- | --- |
| model_HBehav1_reduced | 0 | 0 |
| model_HBehav1_null | -0.4 | 0.7 |
| model_HBehav1 | -1 | 0.4 |

| **Summary**: | Estimate | Est.Error | lower 95% CI | upper 95% CI | Rhat | Bulk_ESS | BF |
| --- | --- | --- | --- | --- | --- | --- | --- |
| Intercept | 0.66 | 0.96 | -1.28 | 2.54 | 1 | 4000 | 0.63 |
| speciesWolf | -0.45 | 0.51 | -1.42 | 0.55 | 1 | 5096 | 1.58 |
| IAT_D1Score.z | 0.1 | 0.43 | -0.75 | 0.92 | 1 | 6891 | 0.86 |
| Explicit.z | 0.34 | 0.43 | -0.47 | 1.17 | 1 | 5674 | 1.08 |
| Experience_years.z | 0.04 | 0.43 | -0.8 | 0.91 | 1 | 6916 | 0.84 |

### Human PC2: Open Talking

**Priors**: prior(normal(0, 0.5), class = "b"), prior(normal(0, 2), class = "Intercept")

model_HBehav2_reduced <- brm(

formula = PC2_Hum_OpenTalking ~ species + (IAT_D1Score.z + Explicit.z) + Experience_years.z + (1 | trainer) +(1|Animal),

data = df,

family = gaussian(),

prior = priors_HBehav2,

chains = 4,

iter = 4000,

control = list(adapt_delta = 0.9),

seed = 1234)

**Full-null model comparison:**

|  | elpd_diff | se_diff |
| --- | --- | --- |
| model_HBehav2_null | 0 | 0 |
| model_HBehav2_reduced | -0.9 | 0.9 |
| model_HBehav2 | -2.2 | 1.1 |

| **Summary**: | Estimate | Est.Error | lower 95% CI | upper 95% CI | Rhat | Bulk_ESS | BF |
| --- | --- | --- | --- | --- | --- | --- | --- |
| Intercept | 0.06 | 0.47 | -0.86 | 0.99 | 1 | 3349 | 0.22 |
| speciesWolf | 0.11 | 0.41 | -0.7 | 0.92 | 1 | 5760 | 0.81 |
| IAT_D1Score.z | -0.05 | 0.22 | -0.47 | 0.41 | 1 | 6415 | 0.48 |
| Explicit.z | -0.11 | 0.22 | -0.53 | 0.35 | 1 | 5699 | 0.56 |
| Experience_years.z | 0.26 | 0.24 | -0.25 | 0.71 | 1 | 5330 | 0.83 |

## Q 3-a: Does human attitude influence animal behavior?

### Animal PC1: Affiliation

**Priors**: prior(normal(0, 0.5), class = "b"), prior(normal(0, 10), class = "Intercept")

model_APC1Att <- brm(

formula = PC1_Anim_Affiliation ~ species * (IAT_D1Score.z + Explicit.z) + Experience_years.z + (1 | trainer) +(1|Animal),

data = df,

family = gaussian(),

prior = priors_APC1Att,

chains = 4, # more MCMC chains improve robustness of posterior

iter = 4000,

control = list(adapt_delta = 0.92), #delta = acceptance rate (default 80%)

seed = 1234,

save_pars = save_pars(all = TRUE))

**Full-null model comparison:**

|  | elpd_diff | se_diff |
| --- | --- | --- |
| model_APC1Att | 0 | 0 |
| model_APC1Att_reduced | -1.2 | 1.3 |
| model_APC1Att_null | -2 | 2 |

| **Summary**: | Estimate | Est.Error | lower 95% CI | upper 95% CI | Rhat | Bulk_ESS | BF |
| --- | --- | --- | --- | --- | --- | --- | --- |
| Intercept | 0.31 | 1.68 | -3.07 | 3.67 | 1 | 2123 | 0.16 |
| speciesWolf | -0.22 | 0.5 | -1.2 | 0.77 | 1 | 9191 | 1.07 |
| IAT_D1Score.z | -0.07 | 0.34 | -0.72 | 0.6 | 1 | 7320 | 0.68 |
| Explicit.z | 0.3 | 0.36 | -0.39 | 1.01 | 1 | 7958 | 1.00 |
| Experience_years.z | 0.41 | 0.35 | -0.32 | 1.06 | 1 | 6782 | 1.52 |
| speciesWolf:IAT_D1Score.z | 0.26 | 0.38 | -0.48 | 0.99 | 1 | 9037 | 0.94 |
| speciesWolf:Explicit.z | 0.62 | 0.41 | -0.18 | 1.41 | 1 | 9106 | 2.66 |

### Animal PC2: Interest

**Priors**: prior(normal(0, 0.5), class = "b"), prior(normal(0, 10), class = "Intercept")

model_APC2Att_reduced <- brm(

formula = PC2_Anim_Interest ~ species + (IAT_D1Score.z + Explicit.z) + Experience_years.z + (1 | trainer) +(1|Animal),

data = df,

family = gaussian(),

prior = priors_APC2Att,

chains = 4,

iter = 4000,

control = list(adapt_delta = 0.93),

seed = 1234)

**Full-null model comparison:**

|  | elpd_diff | se_diff |
| --- | --- | --- |
| model_APC2Att_null | 0 | 0 |
| model_APC2Att_reduced | -0.6 | 1.2 |
| model_APC2Att | -1.2 | 1.6 |

| **Summary**: | Estimate | Est.Error | lower 95% CI | upper 95% CI | Rhat | Bulk_ESS | BF |
| --- | --- | --- | --- | --- | --- | --- | --- |
| Intercept | 0.18 | 0.48 | -0.76 | 1.14 | 1 | 3885 | 0.05 |
| speciesWolf | 0.03 | 0.42 | -0.78 | 0.84 | 1 | 6970 | 0.84 |
| IAT_D1Score.z | -0.03 | 0.26 | -0.53 | 0.47 | 1 | 6437 | 0.55 |
| Explicit.z | 0.13 | 0.28 | -0.44 | 0.67 | 1 | 5948 | 0.61 |
| Experience_years.z | -0.3 | 0.27 | -0.84 | 0.22 | 1 | 6326 | 1.14 |

### Animal PC3: Discomfort

**Priors**: prior(normal(0, 0.5), class = "b"), prior(normal(0, 3), class = "Intercept")

model_APC3Att <- brm(

formula = PC3_Anim_Discomfort ~ species * (IAT_D1Score.z + Explicit.z) + Experience_years.z + (1 | trainer) +(1|Animal),

data = df,

family = gaussian(),

prior = priors_APC3Att,

chains = 4, # more MCMC chains improve robustness of posterior

iter = 4000,

control = list(adapt_delta = 0.93))

**Full-null model comparison:**

|  | elpd_diff | se_diff |
| --- | --- | --- |
| model_APC3Att | 0 | 0 |
| model_APC3Att_null | -0.2 | 1.8 |
| model_APC3Att_reduced | -2.1 | 1.6 |

| **Summary**: | Estimate | Est.Error | lower 95% CI | upper 95% CI | Rhat | Bulk_ESS | BF |
| --- | --- | --- | --- | --- | --- | --- | --- |
| Intercept | -0.12 | 0.47 | -1.05 | 0.84 | 1 | 2869 | 0.15 |
| speciesWolf | 0.1 | 0.41 | -0.71 | 0.91 | 1 | 5478 | 0.82 |
| IAT_D1Score.z | 0.16 | 0.21 | -0.26 | 0.57 | 1 | 6433 | 0.60 |
| Explicit.z | 0.33 | 0.23 | -0.15 | 0.76 | 1 | 5576 | 1.44 |
| Experience_years.z | 0.19 | 0.2 | -0.23 | 0.57 | 1 | 6023 | 0.69 |
| speciesWolf:IAT_D1Score.z | -0.26 | 0.24 | -0.73 | 0.21 | 1 | 8407 | 0.86 |
| speciesWolf:Explicit.z | -0.52 | 0.27 | -1.04 | 0.02 | 1 | 8456 | 3.31 |

## Q 3-b: Does human physiology influence animal behavior?

### Animal PC1: Affiliation

**Priors**: prior(normal(0, 0.5), class = "b"), prior(normal(0, 10), class = "Intercept")

model_APC1_phys <- brm(

formula = PC1_Anim_Affiliation ~ species * (Cort_Change.z + Mean_ChangeHR.z + Mean_ChangeRMSSD_corr1.z) + Experience_years.z + (1 | trainer) +(1|Animal),

data = df,

family = gaussian(),

prior = priors_APC1_phys,

chains = 4, # more MCMC chains improve robustness of posterior

iter = 4000,

control = list(adapt_delta = 0.80), #delta = acceptance rate (default 80%)

seed = 1234,

save_pars = save_pars(all = TRUE))

**Full-null model comparison:**

|  | elpd_diff | | se_diff | |  |  |  |  |  |  |
| --- | --- | --- | --- | --- | --- | --- | --- | --- | --- | --- |
| model_APC1_phys | 0 | | 0 | |  |  |  |  |  |  |
| model_APC1_phys_null | -1.9 | | 2.4 | |  |  |  |  |  |  |
| model_APC1_phys_reduced | -2.8 | | 2 | |  |  |  |  |  |  |
| **Summary**: | | Estimate | | Est.Error | | lower 95% CI | upper 95% CI | Rhat | Bulk_ESS | BF |
| Intercept | | -0.18 | | 1.84 | | -3.8 | 3.43 | 1 | 2003 | 0.18 |
| speciesWolf | | -0.2 | | 0.51 | | -1.21 | 0.78 | 1 | 11209 | 1.14 |
| Cort_Change.z | | -0.01 | | 0.36 | | -0.72 | 0.69 | 1 | 9626 | 0.71 |
| Mean_ChangeHR.z | | 0.27 | | 0.35 | | -0.44 | 0.94 | 1 | 10868 | 0.98 |
| Mean_ChangeRMSSD_corr1.z | | 0.45 | | 0.35 | | -0.26 | 1.14 | 1 | 9837 | 1.64 |
| Experience_years.z | | 0.15 | | 0.33 | | -0.5 | 0.78 | 1 | 10292 | 0.74 |
| speciesWolf:Cort_Change.z | | -0.1 | | 0.38 | | -0.85 | 0.64 | 1 | 9808 | 0.78 |
| speciesWolf:Mean_ChangeHR.z | | 0.85 | | 0.43 | | -0.01 | 1.69 | 1 | 11532 | 6.20 |
| speciesWolf:Mean_ChangeRMSSD  _corr1.z | | -0.02 | | 0.4 | | -0.79 | 0.75 | 1 | 12284 | 0.79 |

### Animal PC2: Interest

**Priors**: prior(normal(0, 0.5), class = "b"), prior(normal(0, 10), class = "Intercept")

model_APC2_phys <- brm(

formula = PC2_Anim_Interest ~ species * (Cort_Change.z + Mean_ChangeHR.z + Mean_ChangeRMSSD_corr1.z) + Experience_years.z + (1 | trainer) +(1|Animal),

data = df,

family = gaussian(),

prior = priors_APC2_phys,

chains = 4, # more MCMC chains improve robustness of posterior

iter = 4000,

control = list(adapt_delta = 0.85), #delta = acceptance rate (default 80%)

seed = 1234,

save_pars = save_pars(all = TRUE))

**Full-null model comparison:**

|  | elpd_diff | se_diff |
| --- | --- | --- |
| model_APC2_phys_reduced | 0 | 0 |
| model_APC2_phys | -0.1 | 0.9 |
| model_APC2_phys_null | -0.3 | 1.6 |

| **Summary**: | Estimate | Est.Error | lower 95% CI | upper 95% CI | Rhat | Bulk_ESS | BF |
| --- | --- | --- | --- | --- | --- | --- | --- |
| Intercept | 0.13 | 0.51 | -0.9 | 1.16 | 1 | 4711 | 0.05 |
| speciesWolf | -0.04 | 0.42 | -0.86 | 0.81 | 1 | 8144 | 0.87 |
| Cort_Change.z | 0.1 | 0.31 | -0.5 | 0.73 | 1 | 7423 | 0.63 |
| Mean_ChangeHR.z | -0.04 | 0.3 | -0.65 | 0.53 | 1 | 7080 | 0.62 |
| Mean_ChangeRMSSD_corr1.z | -0.18 | 0.3 | -0.77 | 0.41 | 1 | 7536 | 0.72 |
| Experience_years.z | -0.27 | 0.26 | -0.78 | 0.26 | 1 | 7604 | 0.91 |
| speciesWolf:Cort_Change.z | 0.31 | 0.34 | -0.38 | 0.98 | 1 | 7588 | 1.09 |
| speciesWolf:Mean_ChangeHR.z | -0.33 | 0.37 | -1.06 | 0.4 | 1 | 8652 | 1.12 |
| speciesWolf:Mean_ChangeRMSSD  _corr1.z | -0.15 | 0.36 | -0.85 | 0.54 | 1 | 7796 | 0.81 |

### Animal PC3: Discomfort

**Priors**: prior(normal(0, 0.5), class = "b"), prior(normal(0, 3), class = "Intercept")

model_APC3_phys <- brm(

formula = PC3_Anim_Discomfort ~ species * (Cort_Change.z + Mean_ChangeHR.z + Mean_ChangeRMSSD_corr1.z) + Experience_years.z + (1 | trainer) +(1|Animal),

data = df,

family = gaussian(),

prior = priors_APC3_phys,

chains = 4, # more MCMC chains improve robustness of posterior

iter = 4000,

control = list(adapt_delta = 0.92), #delta = acceptance rate (default 80%)

seed = 1234,

save_pars = save_pars(all = TRUE))

**Full-null model comparison:**

|  | elpd_diff | se_diff |
| --- | --- | --- |
| model_APC3_phys_null | 0 | 0 |
| model_APC3_phys | -2.5 | 1.7 |
| model_APC3_phys_reduced | -2.8 | 1.4 |

| **Summary**: | Estimate | Est.Error | lower 95% CI | upper 95% CI | Rhat | Bulk_ESS | BF |
| --- | --- | --- | --- | --- | --- | --- | --- |
| Intercept | 0 | 0.48 | -0.96 | 0.99 | 1 | 3636 | 0.15 |
| speciesWolf | 0.07 | 0.42 | -0.76 | 0.89 | 1 | 7183 | 0.83 |
| Cort_Change.z | -0.13 | 0.25 | -0.61 | 0.36 | 1 | 8470 | 0.56 |
| Mean_ChangeHR.z | -0.02 | 0.23 | -0.49 | 0.45 | 1 | 6939 | 0.46 |
| Mean_ChangeRMSSD_corr1.z | -0.36 | 0.24 | -0.83 | 0.12 | 1 | 7161 | 1.52 |
| Experience_years.z | 0.05 | 0.18 | -0.31 | 0.41 | 1 | 8494 | 0.35 |
| speciesWolf:Cort_Change.z | 0.09 | 0.28 | -0.47 | 0.65 | 1 | 8587 | 0.61 |
| speciesWolf:Mean_ChangeHR.z | 0.07 | 0.33 | -0.56 | 0.71 | 1 | 8585 | 0.67 |
| speciesWolf:Mean_ChangeRMSSD  _corr1.z | 0.4 | 0.29 | -0.17 | 0.96 | 1 | 8069 | 1.50 |

# Time series PCA

## Human PCA

**Eigenvalues:**

|  | eigenvalue | Variance (%) | Cumulative variance (%) | |
| --- | --- | --- | --- | --- |
| **Dim.1: PC1-Active Contact** | 1.49 | 22.61 | 22.61 |  |
| **Dim.2: PC2-Open posture** | 1.38 | 20.98 | 43.58 |  |
| **Dim.3: PC3-Direction** | 1.13 | 17.16 | 60.74 |  |
| Dim.4 | 1.01 | 15.27 | 76.02 |  |
| Dim.5 | 0.75 | 11.30 | 87.31 |  |

**Loadings:**

|  | Dim.1 **PC1-Active Contact** | Dim.2 **PC2-**  **Open posture** | Dim.3 **PC3-Direction** |
| --- | --- | --- | --- |
| Active.touching_Aprox0_z | **0.71** | -0.22 | -0.52 |
| BodyAngled_Avis0_z | -0.14 | 0.05 | **0.47** |
| Calling.by.name..S._z | 0.02 | -0.01 | **0.04** |
| Decreasing.distance_Aprox0_z | 0.53 | -0.25 | **0.80** |
| Gazing_Avis0_z | **0.06** | -0.01 | -0.06 |
| Open.Closed_Angled0_z | 0.41 | **1.11** | 0.05 |
| Talking.to.the.animal..S._z | **0.72** | -0.21 | 0.00 |
| Stress_Selfd.ShouldUp.Rep_z | -0.03 | 0.01 | **0.05** |

## Animal PCA

**Eigenvalues:**

|  | eigenvalue | Variance (%) | Cumulative variance (%) |
| --- | --- | --- | --- |
| **Dim.1: PC1-Attention** | 2.15 | 26.84 | 26.84 |
| **Dim.2: PC2-Interest** | 1.45 | 18.10 | 44.93 |
| **Dim.3: PC3-Discomfort** | 1.01 | 12.69 | 57.62 |
| Dim.4 | 1.00 | 12.54 | 70.16 |
| Dim.5 | 0.94 | 11.79 | 81.94 |
| Dim.6 | 0.68 | 8.48 | 90.43 |

**Loadings:**

|  | Dim.1 **PC1-Attention** | Dim.2 **PC2-interest** | Dim.3 **PC3-Discomfort** |
| --- | --- | --- | --- |
| ApproachLeave_Ratio0_z | 0.06 | **0.30** | 0.42 |
| Gazing.at.human_z | **0.85** | 0.26 | -0.06 |
| PhysicalContact_AvisNA_z | **-0.86** | 0.11 | -0.07 |
| Tailwag_whileVis_z | 0.01 | **0.78** | 0.03 |
| Command.followed..T._z | **0.80** | -0.14 | 0.02 |
| Exploring_whileProxNA_z | -0.11 | **0.71** | -0.38 |
| Nose.licks_z | 0.09 | 0.37 | **0.37** |
| Stress_YawnGroomShake_z | -0.17 | 0.01 | **0.74** |

# Time series output

Since the variables showed a non-linear trend (**Figure S3**), we used generalized additive mixed models (GAMMs) controlled for residual autocorrelation.

***Figure S3****:* ***The temporal progression of the three human (H) and three animal (A) behavioral principal components across the two test phases for dogs and wolves respectively.*** *Time is presented in time buckets, with each time segment representing 5s. Note that the cuddle phase was directly followed by the training phase within the data analysis, explaining the sharp change in variable progression at 75 time buckets (5 minutes). The confidence interval around each variable is shown in shaded grey.*

An overview of the time series output can be found in **Figure S4.**

***Figure S4: The time-series-based effects of a) human behavioral variables on animal behavior and b) vice versa.*** *Partial effect of the behavior displayed on the x-axis (lagged by one bin = 5s) on the behavior of the other party on the y-axis five seconds later. Solid lines show smooths for wolves (grey) and dogs (red), with 95% confidence intervals represented as shaded areas. All variables were based on the time-series-based PCA, z-scored, and estimated in a generalized additive mixed model (GAMM) that modelled temporal autocorrelation. Effect sizes, standard deviations and significance for each variable combination are displayed in* ***Table 1****.*

## Human influence on PC1_Animal_Affiliation

**Full model:**

A1H_model_AR_NORMAL <- gam(Dim1_A_Attention.1.z_lag1 ~

Species*(Dim1_H_ActiveContact.z_lag2 + Dim2_H_OpenPosture.z_lag2+ Dim3_H_Direction.z_lag2) +

Experience.z + Phase +

s(Bin.adapted, bs="cr", k=10)

+ s(HumanID, bs="re")+ s(HumanID, Dim1_H_ActiveContact.z_lag2, bs="re")

+ s(HumanID, Dim2_H_OpenPosture.z_lag2, bs="re") + s(HumanID, Dim3_H_Direction.z_lag2, bs="re")

+ s(AnimalID, bs="re")+ s(AnimalID, Dim1_H_ActiveContact.z_lag2, bs="re")

+ s(AnimalID, Dim2_H_OpenPosture.z_lag2, bs="re") + s(AnimalID, Dim3_H_Direction.z_lag2, bs="re")

+ s(DyadID, bs="re") ,

method="ML", data = data_lagged_A1H,

rho=0.2, AR.start = AR.start)

**Full-null model comparison:**

| Model | Score | Edf | Difference | Df | p.value | Sig. |
| --- | --- | --- | --- | --- | --- | --- |
| A1H_gam_NULL | 4369.506 | 14 |  |  |  |  |
| A1H_model_AR_NORMAL | 4349.701 | 21 | 19.804 | 7 | <0.001 | *** |

**Output full model:**

|  | Estimate | Std. Error | t-value | p-value |  |
| --- | --- | --- | --- | --- | --- |
| (Intercept) | 0.62 | 0.17 | 3.71 | 0.00 | *** |
| SpeciesWolf | -0.15 | 0.05 | -2.88 | 0.00 | ** |
| Dim1_H_ActiveContact.z_lag2 | -0.10 | 0.02 | -4.21 | <0.001 | *** |
| Dim2_H_OpenPosture.z_lag2 | 0.33 | 0.08 | 4.04 | <0.001 | *** |
| Dim3_H_Direction.z_lag2 | 0.18 | 0.02 | 7.43 | <0.001 | *** |
| Experience.z | -0.01 | 0.02 | -0.37 | 0.71 |  |
| PhaseTraining | -1.06 | 0.30 | -3.51 | 0.00 | *** |
| SpeciesWolf:Dim1_H_ActiveContact.z_lag2 | -0.01 | 0.04 | -0.22 | 0.83 |  |
| SpeciesWolf:Dim2_H_OpenPosture.z_lag2 | -0.29 | 0.12 | -2.39 | 0.02 | * |
| SpeciesWolf:Dim3_H_Direction.z_lag2 | -0.06 | 0.02 | -2.61 | 0.01 | ** |

**Reduced model:**

A1H_model_AR_REDUCED <- gam(Dim1_A_Attention.1.z_lag1 ~

Species*(Dim2_H_OpenPosture.z_lag2+ Dim3_H_Direction.z_lag2) +

Dim1_H_ActiveContact.z_lag2 +

Experience.z + Phase +

s(Bin.adapted, bs="cr", k=10)

+ s(HumanID, bs="re")+ s(HumanID, Dim1_H_ActiveContact.z_lag2, bs="re")

+ s(HumanID, Dim2_H_OpenPosture.z_lag2, bs="re") + s(HumanID, Dim3_H_Direction.z_lag2, bs="re")

+ s(AnimalID, bs="re")+ s(AnimalID, Dim1_H_ActiveContact.z_lag2, bs="re")

+ s(AnimalID, Dim2_H_OpenPosture.z_lag2, bs="re") + s(AnimalID, Dim3_H_Direction.z_lag2, bs="re")

+ s(DyadID, bs="re") ,

method="ML", data = data_lagged_A1H,

rho=0.2, AR.start = AR.start)

**Output reduced model:**

|  | Estimate | Std. Error | t-value | p-value |  |
| --- | --- | --- | --- | --- | --- |
| (Intercept) | 0.62 | 0.17 | 3.71 | <0.001 | *** |
| SpeciesWolf | -0.15 | 0.05 | -2.87 | <0.001 | ** |
| Dim2_H_OpenPosture.z_lag2 | 0.32 | 0.08 | 4.21 | <0.001 | *** |
| Dim3_H_Direction.z_lag2 | 0.18 | 0.02 | 7.41 | <0.001 | *** |
| Dim1_H_ActiveContact.z_lag2 | -0.10 | 0.02 | -5.06 | <0.001 | *** |
| Experience.z | -0.01 | 0.02 | -0.37 | 0.71 |  |
| PhaseTraining | -1.06 | 0.30 | -3.52 | <0.001 | *** |
| SpeciesWolf:Dim2_H_OpenPosture.z_lag2 | -0.27 | 0.09 | -2.98 | <0.001 | ** |
| SpeciesWolf:Dim3_H_Direction.z_lag2 | -0.06 | 0.02 | -2.60 | 0.01 | ** |

## Human influence on PC2_Animal_Interest

**Full model:**

A2H_model_AR_NORMAL <- gam(Dim2_A_Interest.1.z_lag1 ~

Species*(Dim1_H_ActiveContact.z_lag2 + Dim2_H_OpenPosture.z_lag2+ Dim3_H_Direction.z_lag2) +

Experience.z + Phase +

s(Bin.adapted, bs="cr", k=10)

+ s(HumanID, bs="re")+ s(HumanID, Dim1_H_ActiveContact.z_lag2, bs="re")

+ s(HumanID, Dim2_H_OpenPosture.z_lag2, bs="re") + s(HumanID, Dim3_H_Direction.z_lag2, bs="re")

+ s(AnimalID, bs="re")+ s(AnimalID, Dim1_H_ActiveContact.z_lag2, bs="re")

+ s(AnimalID, Dim2_H_OpenPosture.z_lag2, bs="re") + s(AnimalID, Dim3_H_Direction.z_lag2, bs="re")

+ s(DyadID, bs="re") ,

method="ML", data = data_lagged_A2H,

rho=0.41, AR.start = AR.start)

**Full-null model comparison:**

| Model | Score | Edf | Difference | Df | p.value | Sig. |
| --- | --- | --- | --- | --- | --- | --- |
| A2H_gam_NULL | 6915.186 | 14 |  |  |  |  |
| A2H_model_AR_NORMAL | 6901.829 | 21 | 13.358 | 7 | 3.75E-04 | *** |

**Output full model:**

|  | Estimate | Std. Error | t-value | p-value |  |
| --- | --- | --- | --- | --- | --- |
| (Intercept) | -0.08 | 0.23 | -0.36 | 0.72 |  |
| SpeciesWolf | -0.35 | 0.11 | -3.14 | 0.00 | ** |
| Dim1_H_ActiveContact.z_lag2 | -0.11 | 0.09 | -1.15 | 0.25 |  |
| Dim2_H_OpenPosture.z_lag2 | 0.01 | 0.03 | 0.29 | 0.78 |  |
| Dim3_H_Direction.z_lag2 | 0.24 | 0.07 | 3.53 | 0.00 | *** |
| Experience.z | 0.01 | 0.03 | 0.40 | 0.69 |  |
| PhaseTraining | 0.35 | 0.41 | 0.87 | 0.38 |  |
| SpeciesWolf:Dim1_H_ActiveContact.z_lag2 | -0.09 | 0.12 | -0.72 | 0.47 |  |
| SpeciesWolf:Dim2_H_OpenPosture.z_lag2 | 0.10 | 0.03 | 2.77 | 0.01 | ** |
| SpeciesWolf:Dim3_H_Direction.z_lag2 | -0.10 | 0.09 | -1.10 | 0.27 |  |

**Reduced model:**

A2H_model_AR_REDUCED <- gam(Dim2_A_Interest.1.z_lag1 ~

Species*Dim2_H_OpenPosture.z_lag2 +

Dim1_H_ActiveContact.z_lag2 + Dim3_H_Direction.z_lag2 +

Experience.z + Phase +

s(Bin.adapted, bs="cr", k=10)

+ s(HumanID, bs="re")+ s(HumanID, Dim1_H_ActiveContact.z_lag2, bs="re")

+ s(HumanID, Dim2_H_OpenPosture.z_lag2, bs="re") + s(HumanID, Dim3_H_Direction.z_lag2, bs="re")

+ s(AnimalID, bs="re")+ s(AnimalID, Dim1_H_ActiveContact.z_lag2, bs="re")

+ s(AnimalID, Dim2_H_OpenPosture.z_lag2, bs="re") + s(AnimalID, Dim3_H_Direction.z_lag2, bs="re")

+ s(DyadID, bs="re") ,

method="ML", data = data_lagged_A2H,

rho=0.41, AR.start = AR.start)

**Output reduced model:**

|  | Estimate | Std. Error | t-value | p-value |  |
| --- | --- | --- | --- | --- | --- |
| (Intercept) | -0.08 | 0.19 | -0.45 | 0.65 |  |
| SpeciesWolf | -0.35 | 0.03 | -12.80 | <0.001 | *** |
| Dim2_H_OpenPosture.z_lag2 | 0.01 | 0.02 | 0.23 | 0.82 |  |
| Dim1_H_ActiveContact.z_lag2 | -0.15 | 0.02 | -8.42 | <0.001 | *** |
| Dim3_H_Direction.z_lag2 | 0.19 | 0.01 | 13.26 | <0.001 | *** |
| Experience.z | 0.01 | 0.01 | 0.95 | 0.34 |  |
| PhaseTraining | 0.35 | 0.34 | 1.03 | 0.30 |  |
| SpeciesWolf:Dim2_H_OpenPosture.z_lag2 | 0.10 | 0.03 | 3.03 | 0.00 | ** |

## Human influence on PC3_Animal_Discomfort

**Full model:**

A3H_model_AR_NORMAL <- gam(Dim3_A_Discomfort.1.z_lag1 ~

Species*(Dim1_H_ActiveContact.z_lag2 + Dim2_H_OpenPosture.z_lag2+ Dim3_H_Direction.z_lag2) +

Experience.z + Phase +

s(Bin.adapted, bs="cr", k=10)

+ s(HumanID, bs="re")+ s(HumanID, Dim1_H_ActiveContact.z_lag2, bs="re")

+ s(HumanID, Dim2_H_OpenPosture.z_lag2, bs="re") + s(HumanID, Dim3_H_Direction.z_lag2, bs="re")

+ s(AnimalID, bs="re")+ s(AnimalID, Dim1_H_ActiveContact.z_lag2, bs="re")

+ s(AnimalID, Dim2_H_OpenPosture.z_lag2, bs="re") + s(AnimalID, Dim3_H_Direction.z_lag2, bs="re")

+ s(DyadID, bs="re") ,

method="ML", data = data_lagged_A3H,

rho=0.02, AR.start = AR.start)

**Full-null model comparison:**

| Model | Score | Edf | Difference | Df | p.value | Sig. |
| --- | --- | --- | --- | --- | --- | --- |
| A3H_gam_NULL | 8145.085 | 14 |  |  |  |  |
| A3H_model_AR_NORMAL | 8143.3 | 21 | 1.786 | 7 | 0.828 |  |

**Output full model:**

|  | Estimate | Std. Error | t-value | p-value |  |
| --- | --- | --- | --- | --- | --- |
| (Intercept) | -0.08 | 0.05 | -1.83 | 0.07 | . |
| SpeciesWolf | 0.06 | 0.03 | 1.98 | 0.05 | * |
| Dim1_H_ActiveContact.z_lag2 | -0.06 | 0.02 | -2.49 | 0.01 | * |
| Dim2_H_OpenPosture.z_lag2 | 0.01 | 0.02 | 0.29 | 0.77 |  |
| Dim3_H_Direction.z_lag2 | -0.01 | 0.02 | -0.57 | 0.57 |  |
| Experience.z | 0.01 | 0.01 | 0.60 | 0.55 |  |
| PhaseTraining | 0.08 | 0.08 | 1.05 | 0.29 |  |
| SpeciesWolf:Dim1_H_ActiveContact.z_lag2 | 0.06 | 0.03 | 1.92 | 0.05 | . |
| SpeciesWolf:Dim2_H_OpenPosture.z_lag2 | -0.01 | 0.03 | -0.27 | 0.79 |  |
| SpeciesWolf:Dim3_H_Direction.z_lag2 | -0.05 | 0.03 | -1.76 | 0.08 | . |

## Animal influence on PC1_Human_ActiveContact

**Full model:**

H1H_model_AR_NORMAL <- gam(Dim1_H_ActiveContact.z_lag1 ~

Species*(Dim1_A_Attention.1.z_lag2 + Dim2_A_Interest.1.z_lag2+ Dim3_A_Discomfort.1.z_lag2) +

Experience.z + Phase +

s(Bin.adapted, bs="cr", k=10)

+ s(HumanID, bs="re")+ s(HumanID, Dim1_A_Attention.1.z_lag2, bs="re")

+ s(HumanID, Dim2_A_Interest.1.z_lag2, bs="re") + s(HumanID, Dim3_A_Discomfort.1.z_lag2, bs="re")

+ s(AnimalID, bs="re")+ s(AnimalID, Dim1_A_Attention.1.z_lag2, bs="re")

+ s(AnimalID, Dim2_A_Interest.1.z_lag2, bs="re") + s(AnimalID, Dim3_A_Discomfort.1.z_lag2, bs="re")

+ s(DyadID, bs="re") ,

method="ML", data = data_lagged_H1H,

rho=0.39, AR.start = AR.start)

**Full-null model comparison:**

| Model | Score | Edf | Difference | Df | p.value | Sig. |
| --- | --- | --- | --- | --- | --- | --- |
| H1H_gam_NULL | 6203.761 | 14 |  |  |  |  |
| H1H_model_AR_NORMAL | 6198.612 | 21 | 5.149 | 7 | 0.172 |  |

**Output full model:**

|  | Estimate | Std. Error | t-value | p-value |  |
| --- | --- | --- | --- | --- | --- |
| (Intercept) | 0.65 | 0.05 | 14.01 | <0.001 | *** |
| SpeciesWolf | 0.09 | 0.02 | 3.78 | 0.00 | *** |
| Dim1_A_Attention.1.z_lag2 | -0.01 | 0.02 | -0.85 | 0.39 |  |
| Dim2_A_Interest.1.z_lag2 | 0.06 | 0.01 | 4.25 | <0.001 | *** |
| Dim3_A_Discomfort.1.z_lag2 | 0.04 | 0.01 | 3.30 | 0.00 | *** |
| Experience.z | 0.05 | 0.01 | 5.26 | <0.001 | *** |
| PhaseTraining | -1.18 | 0.08 | -13.92 | <0.001 | *** |
| SpeciesWolf:Dim1_A_Attention.1.z_lag2 | 0.03 | 0.03 | 1.04 | 0.30 |  |
| SpeciesWolf:Dim2_A_Interest.1.z_lag2 | 0.12 | 0.03 | 3.93 | <0.001 | *** |
| SpeciesWolf:Dim3_A_Discomfort.1.z_lag2 | 0.01 | 0.02 | 0.55 | 0.58 |  |

**Reduced model:**

H1H_model_AR_REDUCED <- gam(Dim1_H_ActiveContact.z_lag1 ~

Species*Dim2_A_Interest.1.z_lag2 +

Dim1_A_Attention.1.z_lag2 + Dim3_A_Discomfort.1.z_lag2 +

Experience.z + Phase +

s(Bin.adapted, bs="cr", k=10)

+ s(HumanID, bs="re")+ s(HumanID, Dim1_A_Attention.1.z_lag2, bs="re")

+ s(HumanID, Dim2_A_Interest.1.z_lag2, bs="re") + s(HumanID, Dim3_A_Discomfort.1.z_lag2, bs="re")

+ s(AnimalID, bs="re")+ s(AnimalID, Dim1_A_Attention.1.z_lag2, bs="re")

+ s(AnimalID, Dim2_A_Interest.1.z_lag2, bs="re") + s(AnimalID, Dim3_A_Discomfort.1.z_lag2, bs="re")

+ s(DyadID, bs="re") ,

method="ML", data = data_lagged_H1H,

rho=0.39, AR.start = AR.start)

**Full-reduced model comparison:**

| Model | Score | Edf | Difference | Df | p.value | Sig. |
| --- | --- | --- | --- | --- | --- | --- |
| H1H_gam_NULL | 6203.761 | 14 |  |  |  |  |
| H1H_model_AR_REDUCED | 6198.734 | 19 | 5.028 | 5 | 0.074 |  |

**Output reduced model:**

|  | Estimate | Std. Error | t-value | p-value |  |
| --- | --- | --- | --- | --- | --- |
| (Intercept) | 0.65 | 0.05 | 14.08 | <0.001 | *** |
| SpeciesWolf | 0.10 | 0.02 | 3.82 | 0.00 | *** |
| Dim2_A_Interest.1.z_lag2 | 0.06 | 0.01 | 4.25 | <0.001 | *** |
| Dim1_A_Attention.1.z_lag2 | 0.00 | 0.02 | -0.15 | 0.88 |  |
| Dim3_A_Discomfort.1.z_lag2 | 0.05 | 0.01 | 4.64 | <0.001 | *** |
| Experience.z | 0.05 | 0.01 | 5.25 | <0.001 | *** |
| PhaseTraining | -1.18 | 0.08 | -13.99 | <0.001 | *** |
| SpeciesWolf:Dim2_A_Interest.1.z_lag2 | 0.13 | 0.03 | 3.97 | <0.001 | *** |

## Animal influence on PC2_Human_OpenPosture

**Full model:**

H2H_model_AR_NORMAL <- gam(Dim2_H_OpenPosture.z_lag1 ~

Species*(Dim1_A_Attention.1.z_lag2 + Dim2_A_Interest.1.z_lag2+ Dim3_A_Discomfort.1.z_lag2) +

Experience.z + Phase +

s(Bin.adapted, bs="cr", k=10)

+ s(HumanID, bs="re")+ s(HumanID, Dim1_A_Attention.1.z_lag2, bs="re")

+ s(HumanID, Dim2_A_Interest.1.z_lag2, bs="re") + s(HumanID, Dim3_A_Discomfort.1.z_lag2, bs="re")

+ s(AnimalID, bs="re")+ s(AnimalID, Dim1_A_Attention.1.z_lag2, bs="re")

+ s(AnimalID, Dim2_A_Interest.1.z_lag2, bs="re") + s(AnimalID, Dim3_A_Discomfort.1.z_lag2, bs="re")

+ s(DyadID, bs="re") ,

method="ML", data = data_lagged_H2H,

rho=0.02, AR.start = AR.start)

**Full-reduced model comparison:**

| Model | Score | Edf | Difference | Df | p.value | Sig. |
| --- | --- | --- | --- | --- | --- | --- |
| H2H_gam_NULL | 8203.683 | 14 |  |  |  |  |
| H2H_model_AR_NORMAL | 8195.881 | 21 | 7.803 | 7 | 0.029 | * |

**Output full model:**

|  | Estimate | Std. Error | t-value | p-value |  |
| --- | --- | --- | --- | --- | --- |
| (Intercept) | -0.18 | 0.05 | -3.84 | 0.00 | *** |
| SpeciesWolf | 0.02 | 0.03 | 0.62 | 0.54 |  |
| Dim1_A_Attention.1.z_lag2 | -0.01 | 0.02 | -0.62 | 0.53 |  |
| Dim2_A_Interest.1.z_lag2 | -0.04 | 0.02 | -2.41 | 0.02 | * |
| Dim3_A_Discomfort.1.z_lag2 | -0.01 | 0.02 | -0.85 | 0.39 |  |
| Experience.z | -0.04 | 0.01 | -3.23 | 0.00 | ** |
| PhaseTraining | 0.33 | 0.09 | 3.83 | 0.00 | *** |
| SpeciesWolf:Dim1_A_Attention.1.z_lag2 | -0.06 | 0.03 | -1.98 | 0.05 | * |
| SpeciesWolf:Dim2_A_Interest.1.z_lag2 | -0.05 | 0.04 | -1.39 | 0.16 |  |
| SpeciesWolf:Dim3_A_Discomfort.1.z_lag2 | -0.02 | 0.03 | -0.69 | 0.49 |  |

**Reduced model:**

H2H_model_AR_REDUCED <- gam(Dim2_H_OpenPosture.z_lag1 ~

Species*(Dim1_A_Attention.1.z_lag2) +

Dim2_A_Interest.1.z_lag2+ Dim3_A_Discomfort.1.z_lag2 +

Experience.z + Phase +

s(Bin.adapted, bs="cr", k=10)

+ s(HumanID, bs="re")+ s(HumanID, Dim1_A_Attention.1.z_lag2, bs="re")

+ s(HumanID, Dim2_A_Interest.1.z_lag2, bs="re") + s(HumanID, Dim3_A_Discomfort.1.z_lag2, bs="re")

+ s(AnimalID, bs="re")+ s(AnimalID,Dim1_A_Attention.1.z_lag2, bs="re")

+ s(AnimalID, Dim2_A_Interest.1.z_lag2, bs="re") + s(AnimalID, Dim3_A_Discomfort.1.z_lag2, bs="re")

+ s(DyadID, bs="re") ,

method="ML", data = data_lagged_H2H,

rho=0.02, AR.start = AR.start)

**Output reduced model:**

|  | Estimate | Std. Error | t-value | p-value |  |
| --- | --- | --- | --- | --- | --- |
| (Intercept) | -0.19 | 0.05 | -4.01 | <0.001 | *** |
| SpeciesWolf | 0.03 | 0.03 | 0.90 | 0.37 |  |
| Dim1_A_Attention.1.z_lag2 | -0.02 | 0.02 | -0.74 | 0.46 |  |
| Dim2_A_Interest.1.z_lag2 | -0.05 | 0.01 | -3.12 | 0.00 | ** |
| Dim3_A_Discomfort.1.z_lag2 | -0.02 | 0.01 | -1.80 | 0.07 | . |
| Experience.z | -0.04 | 0.01 | -3.18 | 0.00 | ** |
| PhaseTraining | 0.34 | 0.09 | 4.03 | <0.001 | *** |
| SpeciesWolf:Dim1_A_Attention.1.z_lag2 | -0.06 | 0.03 | -1.99 | 0.05 | * |

## Animal influence on PC3_Human_Direction

**Full model:**

H3H_model_AR_NORMAL <- gam(Dim3_H_Direction.z_lag1 ~

Species*(Dim1_A_Attention.1.z_lag2 + Dim2_A_Interest.1.z_lag2+ Dim3_A_Discomfort.1.z_lag2) +

Experience.z + Phase +

s(Bin.adapted, bs="cr", k=10)

+ s(HumanID, bs="re")+ s(HumanID, Dim1_A_Attention.1.z_lag2, bs="re")

+ s(HumanID, Dim2_A_Interest.1.z_lag2, bs="re") + s(HumanID, Dim3_A_Discomfort.1.z_lag2, bs="re")

+ s(AnimalID, bs="re")+ s(AnimalID, Dim1_A_Attention.1.z_lag2, bs="re")

+ s(AnimalID, Dim2_A_Interest.1.z_lag2, bs="re") + s(AnimalID, Dim3_A_Discomfort.1.z_lag2, bs="re")

+ s(DyadID, bs="re") ,

method="ML", data = data_lagged_H3H,

rho=0.30, AR.start = AR.start)

**Full-reduced model comparison:**

| Model | Score | Edf | Difference | Df | p.value | Sig. |
| --- | --- | --- | --- | --- | --- | --- |
| H3H_gam_NULL | 7242.236 | 14 |  |  |  |  |
| H3H_model_AR_NORMAL | 7231.67 | 21 | 10.567 | 7 | 0.004 | ** |

**Output full model:**

|  | Estimate | Std. Error | t-value | p-value |  |
| --- | --- | --- | --- | --- | --- |
| (Intercept) | -0.09 | 0.12 | -0.77 | 0.44 |  |
| SpeciesWolf | 0.74 | 0.04 | 19.82 | <0.001 | *** |
| Dim1_A_Attention.1.z_lag2 | 0.32 | 0.02 | 15.25 | <0.001 | *** |
| Dim2_A_Interest.1.z_lag2 | -0.07 | 0.02 | -4.16 | <0.001 | *** |
| Dim3_A_Discomfort.1.z_lag2 | 0.01 | 0.01 | 0.94 | 0.35 |  |
| Experience.z | -0.06 | 0.01 | -4.95 | <0.001 | *** |
| PhaseTraining | -0.04 | 0.23 | -0.19 | 0.85 |  |
| SpeciesWolf:Dim1_A_Attention.1.z_lag2 | -0.19 | 0.03 | -6.03 | <0.001 | *** |
| SpeciesWolf:Dim2_A_Interest.1.z_lag2 | 0.45 | 0.04 | 11.52 | <0.001 | *** |
| SpeciesWolf:Dim3_A_Discomfort.1.z_lag2 | -0.03 | 0.02 | -1.46 | 0.15 |  |

**Reduced model:**

H3H_model_AR_REDUCED <- gam(Dim3_H_Direction.z_lag1 ~

Species*(Dim1_A_Attention.1.z_lag2 + Dim2_A_Interest.1.z_lag2) +

Dim3_A_Discomfort.1.z_lag2 +

Experience.z + Phase +

s(Bin.adapted, bs="cr", k=10)

+ s(HumanID, bs="re")+ s(HumanID, Dim1_A_Attention.1.z_lag2, bs="re")

+ s(HumanID, Dim2_A_Interest.1.z_lag2, bs="re") + s(HumanID, Dim3_A_Discomfort.1.z_lag2, bs="re")

+ s(AnimalID, bs="re")+ s(AnimalID, Dim1_A_Attention.1.z_lag2, bs="re")

+ s(AnimalID, Dim2_A_Interest.1.z_lag2, bs="re") + s(AnimalID, Dim3_A_Discomfort.1.z_lag2, bs="re")

+ s(DyadID, bs="re") ,

method="ML", data = data_lagged_H3H,

rho=0.30, AR.start = AR.start)

**Output reduced model:**

|  | Estimate | Std. Error | t-value | p-value |  |
| --- | --- | --- | --- | --- | --- |
| (Intercept) | -0.10 | 0.12 | -0.82 | 0.41 |  |
| SpeciesWolf | 0.74 | 0.04 | 19.79 | <0.001 | *** |
| Dim1_A_Attention.1.z_lag2 | 0.33 | 0.02 | 15.29 | <0.001 | *** |
| Dim2_A_Interest.1.z_lag2 | -0.07 | 0.02 | -4.23 | <0.001 | *** |
| Dim3_A_Discomfort.1.z_lag2 | 0.00 | 0.01 | 0.09 | 0.93 |  |
| Experience.z | -0.06 | 0.01 | -4.95 | <0.001 | *** |
| PhaseTraining | -0.03 | 0.23 | -0.13 | 0.90 |  |
| SpeciesWolf:Dim1_A_Attention.1.z_lag2 | -0.19 | 0.03 | -6.03 | <0.001 | *** |
| SpeciesWolf:Dim2_A_Interest.1.z_lag2 | 0.45 | 0.04 | 11.45 | <0.001 | *** |
| PhaseTraining | -0.03 | 0.23 | -0.13 | 0.90 |  |
| SpeciesWolf:Dim1_A_Attention.1.z_lag2 | -0.19 | 0.03 | -6.03 | <0.001 | *** |
| SpeciesWolf:Dim2_A_Interest.1.z_lag2 | 0.45 | 0.04 | 11.45 | <0.001 | *** |

**SI References**

1. Greenwald, A. G., Nosek, B. A., & Banaji, M. R. (2003). Understanding and using the implicit association test: I. An improved scoring algorithm. *Journal of personality and social psychology*, *85*(2), 197.
